# Channel Selection Based on Trust and Multiarmed Bandit in Multiuser, Multichannel Cognitive Radio Networks

**DOI:** 10.1155/2014/916156

**Published:** 2014-02-23

**Authors:** Fanzi Zeng, Xinwang Shen

**Affiliations:** Key Laboratory for Embedded and Network Computing of Hunan Province, Hunan University, Changsha 410012, China

## Abstract

This paper proposes a channel selection scheme for the multiuser, multichannel cognitive radio networks. This scheme formulates the channel selection as the multiarmed bandit problem, where cognitive radio users are compared to the players and channels to the arms. By simulation negotiation we can achieve the potential reward on each channel after it is selected for transmission; then the channel with the maximum accumulated rewards is formally chosen. To further improve the performance, the trust model is proposed and combined with multi-armed bandit to address the channel selection problem. Simulation results validate the proposed scheme.

## 1. Introduction

Radio spectrum is known to be a kind of valuable and limited resource. With the explosive growth in wireless applications, radio spectrum resources are almost exhausted. However, reliably experiments show that current spectrum is underutilized and that there exists spectrum opportunity over the space and time. To improve the utilization efficiency of spectrum, dynamic spectrum access (DSA) model based on cognitive radios [1] has been proposed, which allows secondary users (SUs) to exploit the spectrum opportunistically without interfering with primary users (PUs). In this paper, we focus on the critical issue of DSA in cognitive radio networks (CRNs): efficient channel selection strategy.

There exit many pieces of work on DSA strategy, most of which deal with spectrum sensing and channel sharing. In [1–3], the DSA strategies maximize the throughput of a single secondary user in a multichannel slotted primary network. But, there are always multiple secondary users seeking spectrum hole under licensed primary users in CRNs. So, it is very necessary and natural to address the problem of multiuser spectrum sharing in multichannel CRNs. For multi-channel CRNs, every secondary user should decide which channel must be selected to achieve highest throughput. Meanwhile, when multiple secondary users are contending for spectrum opportunities, they should take the best channel without colliding with other users. Effective channel selection for centralized and distributed system has been addressed extensively in the last decade. It is worth mentioning that for a centralized scheme, the communications between the fusion center and secondary users will raise the system reliability, but it also results in increasing the burden of the overall system, while the distributed scheme with lower complexity but no information exchange about the channel selection may lead to collisions among secondary users for some of them choosing the same channel.

In [4, 5], the authors analyzed the CRNs throughput using random sensing with negotiation, but it required an extra control channel for coordination among SUs. Hoang et al. [6] considered centralized power control and channel allocation in order to maximize the total throughput in CRNs, where each user requires the knowledge of all channel gains between PUs and SUs; in that way, it means a great deal of cooperation between PUs and SUs. The same problem has been introduced while jointly considering the result of spectrum sensing in [7]. Some papers have accrued interest in spectrum optimization for frequency selective channels (see [8, 9] and the reference therein). However, all of those pieces of work rely on central fusion controlling channel state information and statistics. In contrast, Li et al. [10] applied analytical results for throughput for a novel slotted ALOHA-based distributed access CR system in a proposed channel selection scheme by considering the relationship between the system throughput and the number of the sensing channels. In [11], Leshem et al. suggested Gale-Shapley stable marriage theorem from game theory into channel allocation, where the user and the channel achieve the stable allocation in the cost of high complexaity. A channel-and-sensing-aware channel access (CSCA) policy is proposed for multichannel interweaves cognitive radio systems in [12], in which a minimum channel gain threshold is set on each channel to limit the probability that the channel is accessed by SUs. Therefore, we can draw a conclusion that if the SU chooses one or a subset of possible channels to access, it is crucial for DSA to design an efficient channel selection strategy to find the best channel. This problem can be formulated as a multi-armed bandit (MAB) problem (has been applied in [13, 14]). The paper [15] used the PAC-MAB formulation with optimal active sensing, which enables the SU to optimally balance energy between sensing and probing and data transmission. This motivates our present work where we transfer the channel selection problem into a MAB problem. Considering that cognitive radio systems are inherently distributed, in this paper, we consider a distributed solution to maximize the total throughput in the multiuser, multichannel CRNs.

The remainder of the paper is organized as follows. The system model and some basic definitions and assumptions used in this paper are presented in Section 2. In Section 3, we solve the problem of determining the optimal access cardinalities based on trust and MAB formulation.  Section 4 is the numerical results of our algorithm. Finally, in Section 5, we conclude this paper and provide future work.

## 2. Problem Formulations

Suppose that there exist *M* secondary users (SUs), *N* available channels. Let ∑_*B*_{*b*_1_, *b*_2_,…*b*_*M*_} denote the SU set and let ∑_*S*_{*s*_1_, *s*_2_,…*s*_*N*_} denote the channels set. The channel selection problem is that SU chooses the suitable channel from the available channel list to transmit to maximize the total channel utility. It is assumed that each SU can select only one channel and access to different channel can obtain different gain. If ∑_*B*_ is compared to the players and ∑_*S*_ to the arms, the channel selection problem falls into the multi-arm bandit problem framework.

For the classical multi-armed bandit problems, the player repeatly selects an arm among a number of alternative arms and observes its associated reward to find an arm that maximizes the average cumulative reward, as close as possible to the ideal reward obtained if we were to try the “best” arm at all times.

Let *e*_*i*,*j*_ denote channel transmission efficiency that *i*th SU selects *j*th channel to transmit,

(1) $$\begin{array}{c} e_{i,j}=t_{i,j}\cdot p_{i,j}\cdot w_{j}\cdot \text{lo}{\text{g}}_{2}\left(1+\frac{\rho_{i,j}\cdot h_{i,j}}{N_{0}}\right), \end{array}$$

where *t*_*i*,*j*_ is the transmission duration of *i*th SU on *j*th channel before collision occurs; *p*_*i*,*j*_ is the probability of the *i*th SU to access the channel *j*; *w*_*j*_  is the bandwidth of channel *j*; *ρ*_*i*,*j*_ = *ρ* · *N*_*i*_ · *d*_*i*_ is the transmission power of the *i*th SU on channel *j*, *ρ* is the transmission power, *N*_*i*_  is the data packet size that *i*th SU wants to transmit, *d*_*i*_ represents the distance between *i*th SU and its receiver; *h*_*i*,*j*_ is the transmission coefficient of channel *j* chosen by ith SU and *N*_0_ is the power spectral density of noise.

Suppose each SU maintains a list it contains the channel transmission efficiency of all channels that have been selected. For ∀*s*_*i*_ ∈ Σ_*s*_, let *H* ^*s*_*i*_^ = (*e*_1_^*s*_*i*_^, *e*_2_^*s*_*i*_^,…*e*_*n*_*i*__^*s*_*i*_^) denote the channel decision history, transmission efficiency of the simulated selection channel when simulated negotiation can be expressed as ${\hat{H}}^{s_{i}}=({\hat{e}}_{1}^{s_{i}},{\hat{e}}_{2}^{s_{i}},\ldots {\hat{e}}_{n_{i}}^{s_{i}})$, where *n*_*i*_ is the number of times channel *s*_*i*_ selected, where *e*_*j*_^*s*_*i*_^ is the transmission efficiency gain after transmission, while ${\hat{e}}_{j}^{s_{i}}$ is transmission efficiency gain achieved by simulated negotiation.

## 3. Channel Selection Based on Multiarmed Bandit Problem

In above section, we formulate channel selection problem as MAB, where SU is compared to players and channels to arms. The player's purpose in MAB system is to maximize his total reward over a sequence of trials. Since each arm is assumed to have a different distribution of reward, the goal is to find the arm with the best expected return. The ultimate objective is to find the reward on each arm; this problem can be solved by simulated negotiation. Therefore, we can apply the algorithm of simulated negotiation into CRNs to obtain channel's reward distribution. In the condition of unknown channel's characteristics, the CR user tries to choose the best channel based on its data characteristic and decision history.

CR user chooses the negotiation target according to the probability *p*_*i*,*j*_ of the *i*th SU to access the channel *j*, and let the channel transmission efficiency gain between the simulation negotiation and the precedent efficiency record denote the interaction reward; that is,

(2) $$\begin{array}{c} \Delta_{j}^{s_{i}}={\hat{e}}_{j}^{s_{i}}-e_{j}^{s_{i}}; \end{array}$$

then, the reward of selecting channel *s*_*i*_ at slot *n* is

(3) $$\begin{array}{c} r^{s_{i}}\left(n\right)=\overset{n_{i}}{\underset{j=1}{\sum }}\Delta_{j}^{s_{i}}. \end{array}$$

Let *R* ^*s*_*i*_^(*n*) denote the total simulation negotiation reward of selecting channel *s*_*i*_ at slot *n*, which can be updated as follows:

(4) $$\begin{array}{c} R^{s_{i}}\left(n\right)=R^{s_{i}}\left(n-1\right)+r^{s_{i}}\left(n\right). \end{array}$$

Typically, to solve the MAB problem, the reward on each arm is generated by some statistical assumptions for some circumstance, the distribution of each arm's reward is assumed to be a Gauss and time-invariant. But in fact, it is difficult or even impossible to determine the right statistical assumptions. In this paper, we use the nonstatistical assumptions technique to solve this classic MAB problem. In order to achieve each arm's reward distribution, based on algorithm of Hedge(*β*) discussed in the literature [16] with the simulation of negotiation, apply the MAB technique into the channel selection algorithm to acquire each channel's reward distribution.

Let *α* > 0  be the parameter to determine the probability of selecting the channel *s*_*i*_.

The algorithm is as follows.

*Initialization*. for  *i* = 1,2 … *N*,  *R* ^*s*_*i*_^(1) = 0

Then for *i* = 1, 2 … *N*  do the following:

(1) According to the total simulation negotiation reward to calculate the probability of selecting channel *s*_*i*_ is *p*_*i*_(*n*):
  (5) $$\begin{array}{c} p_{i}\left(n\right)=\frac{{\left(1+\alpha \right)}^{R^{s_{i}}(n)}}{\sum_{j=1}^{N}{\left(1+\alpha \right)}^{R^{s_{j}}(n)}}. \end{array}$$
(2) Opt the channel with the maximum *p*_*i*_(*n*) as the next negotiation object; then, through simulation calculate the reward *r* ^*s*_*i*_^(*n*) of selecting channel *s*_*i*_ at slot *n*.
(3) Update the accumulated reward of channel *s*_*i*_:
  (6) $$\begin{array}{c} R^{s_{i}}\left(n\right)=R^{s_{i}}\left(n-1\right)+r^{s_{i}}\left(n\right)\text{.} \end{array}$$
  Then choose the channel S*:
  (7) $$\begin{array}{c} S^{*}=\underset{1\leq i\leq N}{\arg\;\max}\left(\overset{N}{\underset{j=1}{\sum }}\;p_{i}\left(j\right)r^{s_{i}}\left(j\right)\right). \end{array}$$

However, this algorithm is defective; there may be a channel with a larger reward but its probability of being selected is relatively low. Therefore, we combine the probability distribution *p*_*i*_(*n*) generated by the above algorithm with a uniform distribution to form the new probability distribution $\hat{p_{i}}\left(n\right)$. At the same time, reevaluate each channel's interactive reward to ensure the channel that owns greater rewards and has greater probability to be opted.

Consider the following:

(8) $$\begin{array}{c} {\hat{p}}_{i}\left(n\right)=\left(1-\gamma \right)p_{i}\left(n\right)+\frac{\gamma }{N},\\ {\hat{r}}^{s_{i}}\left(n\right)=\left(\frac{\gamma }{N}\right)\times \frac{r^{s_{i}}\left(n\right)}{\hat{p_{i}}\left(n\right)}. \end{array}$$

We can see that accumulated reward ${\hat{r}}^{s_{i}}\left(n\right)$ is proportional to $r^{s_{i}}\left(n\right)/{\hat{p}}_{i}\left(n\right)$, and the expected reward is also proportional to the actual reward; that is, $E\left[{\hat{r}}^{s_{i}}\left(n\right)\right]=\left(\gamma /N\right)\times r^{s_{i}}\left(n\right)$; the expression of *γ*/*N* can guarantee ${\hat{r}}^{s_{i}}\left(n\right)\in \left[0,1\right]$. According to Lemma 4.2 introduced in the reference [17] can determine the parameters *α*, *γ*.

### 3.1. Channel Selection Based on Trust and Multiarmed Bandit

Through the simulated negotiation and updating the cumulative interaction reward, eventually, the user finds the best transmission channels. However, the following conditions will affect the accuracy and validity of the above algorithm: (a) before slot *n*, some channel may not be selected by SU which causes the negotiation history of these channels empty. Thus we cannot use (2) to calculate the interaction reward; (b) the negotiation history is too obsolete to present the current channel characteristic. Thus, in order to further improve efficiency and practicability of channel selection, in this paper, we introduce the trust model into multi-armed bandit problem to propose a channel selection algorithm.

Let *u*_*i*,*j*_ denote channel utilization efficiency that *j*th SU transmit on *i*th channel, and *u*_*i*,*j*_ = *N*_*i*,*j*_/*U*_*i*,*j*_, where *N*_*i*,*j*_ is the number of transmitted packets and *U*_*i*,*j*_ is the channel utilization of the *j*th SU transmit on *i*th channel, which has been solved in [18].

**Definition 1 (belief *B*_*b*→*s*_)**

Before the slot *t*, if the sensor *b* has finished *k* (*k* ≥ 1) times transmission on channel *s* and the channel utilization efficiencies are *u*_1_, *u*_2_,…, *u*_*k*_, where the maximum utilization are *u*_1_*, *u*_2_*,…, *u*_k_*, then the belief of the user on channel *s* is

(9) Bb→s=∑i=1ku  i  ∑i=1kui∗.

**Definition 2 (reputation *R*_*b*→*s*_)**

Before the slot *t*, if sensor *b* hasnot transmitted on channel *s*, we can achieve belief from other users, named sensor's reputation. It is the average of the whole sensor's belief on channel *s* except sensor *b*, which can be expressed as

(10) $$\begin{array}{c} R_{b\to s}=\overset{L}{\underset{i=1,b_{i}}{\sum }}r_{i}^{b}B_{b_{i}\to s}, \end{array}$$

where *L* is the total number of sensors ever transmitted on channel *s*,  *r*_*i*_^*b*^ is the degree of trust of the sensor *b* on other sensor *b*_*i*_, and ∑_*i*=1_^*L*^ *r*_*i*_^*b*^ = 1. In this paper, we assume that *r*_1_^*b*^ = *r*_2_^*b*^ = ⋯*r*_*L*_^*b*^ = 1/*L*.

So, the trust on channel *s* is *T*_*b*→*s*_ = *B*_*b*→*s*_ + *R*_*b*→*s*_. Therefore, we'll combine trust-reputation model with reward distribution to solve best channel selection problem. Firstly, combine trust model with the interactive reward distribution to achieve the probability distribution of each channel; then, apply the average distribution into the channel's probability distribution; next, calculate the trust with the interactive reward and update the channel interactive reward; finally, select the appropriate negotiation target.

The outline of the algorithm is as follows.

*Initialization*. for  *i* = 1,2 … *N*,  ∑_*j*=1_^*N*^ *p*_*i*_(*j*)*r* ^*s*_*i*_^(*j*) = 0  

Then for *j* = 1, 2 … *N* do the following:

(1) Combine trust model with the interactive reward distribution to achieve the probability distribution of each channel.
  For  *j* = 1,2 ⋯ *N*  do the following:
  (11) $$\begin{array}{c} p_{j}\left(n\right)=\frac{{\left(1+\alpha \right)}^{R^{s_{j}}(n)}}{\sum_{i=1}^{N}{\left(1+\alpha \right)}^{R^{s_{i}}(n)}},\\ {\hat{p}}_{j}\left(n\right)={\hat{p}}_{j}\left(n\right)B_{b_{i}\to s_{j}}. \end{array}$$
(2) Apply the average distribution into the channel's probability distribution as follows:
  (12) $$\begin{array}{c} {\hat{p}}_{j}\left(n\right)=\left(1-\gamma \right)p_{j}\left(n\right)+\frac{\gamma }{N},\\ {\hat{r}}^{s_{i}}\left(n\right)=\left(\frac{\gamma }{N}\right)\times \frac{r^{s_{i}}\left(n\right)}{{\hat{p}}_{j}\left(n\right)}. \end{array}$$
(3) Calculate the trust vector with the interactive reward as follows:
  (13) $$\begin{array}{c} {\hat{r}}^{s_{j}}\left(n\right)={\hat{r}}^{s_{j}}\left(n\right)\cdot T_{b\to s}. \end{array}$$
(4) Update accumulative interactive reward of channel *s*_*j*_ as follows:
  (14) $$\begin{array}{c} R^{s_{j}}\left(n\right)=R^{s_{j}}\left(n-1\right)+r^{s_{j}}\left(n\right). \end{array}$$
(5) Select the best channel:
  (15) $$\begin{array}{c} S^{*}=\underset{1\leq i\leq N}{\arg\;\max}\left(\overset{N}{\underset{j=1}{\sum }}\;{\hat{p}}_{i}\left(j\right){\hat{r}}^{s_{i}}\left(j\right)\right). \end{array}$$

## 4. Simulation Results

In this section, we study the performance of the channel selection policy through simulation. 30 SUs associated with 40 channels are located within a circular ring area with radii between 200 m and 1 km, and the transmission coefficient *h*_*i*,*j*_ is randomly selected in 0~1, but unchanged in the whole process of simulation. We run the simulation for 1000  times under the same conditions and each time our algorithm runs for 10000-time slots.

**Figure 1:**
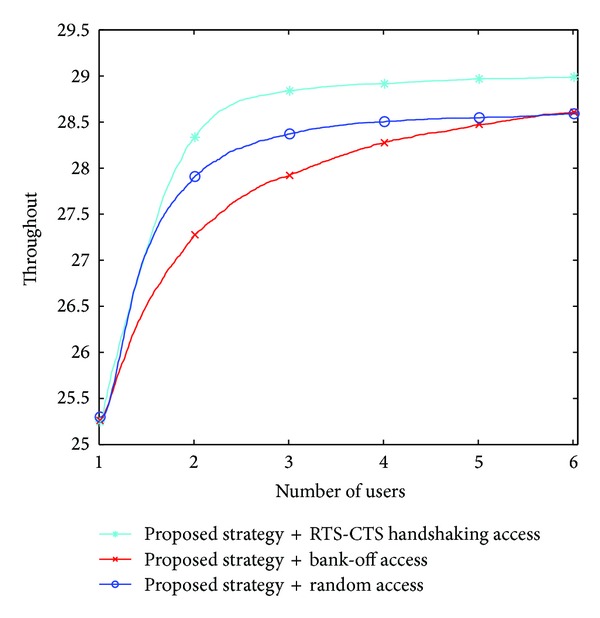
System throughput under different access strategy.

**Figure 2:**
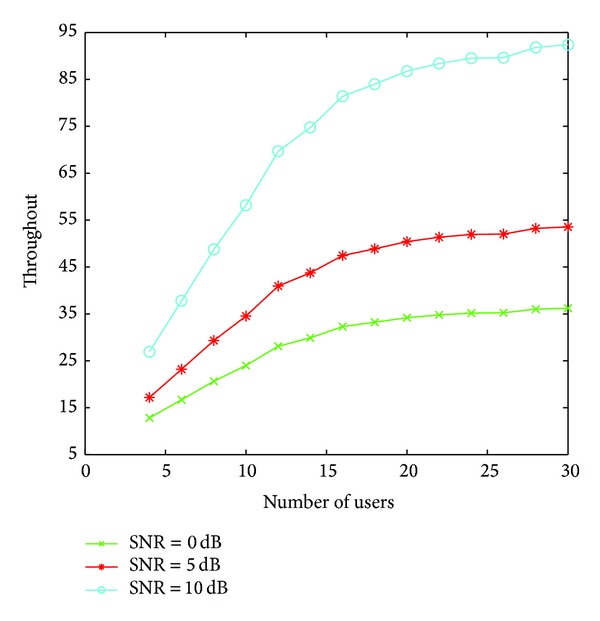
System throughput under different SNR condition.

**Figure 3:**
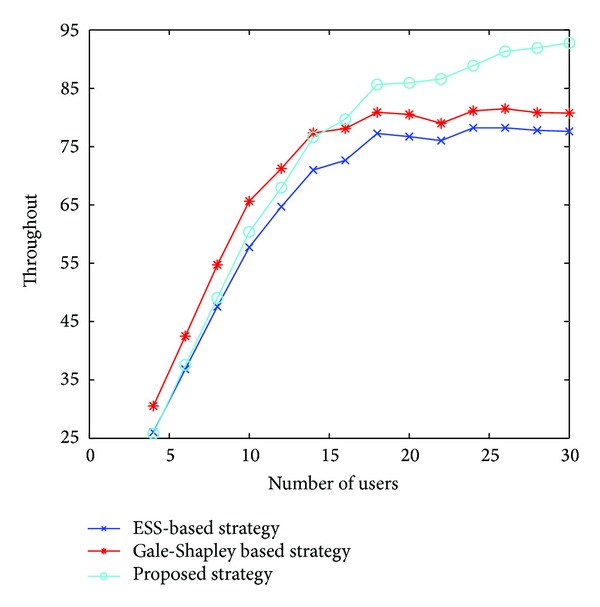
System throughput under different channel selection strategies.

**Figure 4:**
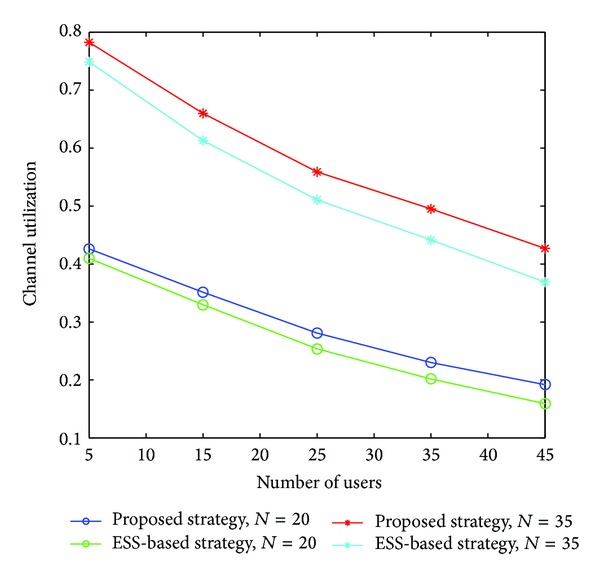
Comparison of channel utilization.

1. When two or more than two users select a channel at the same time, it will lead to conflict. There are two methods to solve this problem: the back-off access method (all of the users quit the channel and find a new one) and random access method. In this paper, we apply RTS-CTS handshaking access; each user sends the preaccess signal before access in. The throughput curves of different access policies are shown in Figure 1. The RTS-CTS handshaking access achieves better throughput than the back-off access and the random access because it can avoid collisions by sending preaccess signal when the two users choose the same channel. Therefore, we jointly consider the proposed strategy and RTS-CTS handshaking access so as to improve the system throughput.
2. We now turn to test the gain of the proposed strategy under different SNR conditions, as it is depicted in Figure 2. Note that for higher values of SNR the gain of the users is higher. The reason is that the good channel that is selected can be fully used for transmission.
3. Here we compare the performance of three strategies in Figure 3: the strategy in [10], named ESS-based strategy, our proposed strategy, and the Gale-Shapley based strategy proposed in [11]. The results in the figure show the superiority of the proposed algorithm. It is noted that the system throughput first increases with the number of users, however, comes to saturation after a limited number of users. This occurs because the limited channel resource cannot bring more access opportunities; on the contrary, sometimes it may bring more collisions results in decreasing throughput.
4. In Figure 4, we compare the channel utilization between ESS-based strategy and our proposed strategy with fixed *M*. We observe that the channel utilization degrades as the number of users increase. One potential cause is that along with the increase of the number of users, the conflict will increase and cause the waste of idle channel. We can also observe that our proposed scheme is more efficient than ESS-based strategy.

## 5. Conclusion

Multi-channel for multi-user to access in is a complicated course. In this paper, we proposed a distributed channel selection strategy based on the combination of trust-reputation model and multiarmed problem policies. Depending on the knowledge of the local observation and history decisions, a relatively efficient and channel selection strategy was obtained with the goal of maximizing the system throughput.

We provide numerical results with different scenarios regarding the system throughput; we show that higher values of SNR the gain higher throughput and the RTS-CTS handshaking access achieves better throughput than the back-off access and the random access. Furthermore, we compare our proposed channel selection algorithm with ESS-based and Gale-Shapley based strategy; simulation results show that this strategy performs better than the other two methods do in throughput gains or time overhead. As we all know, there exists a close relationship between the spectrum sensing and spectrum access; note that we did not consider this problem, which will be one of our future pieces of work.
